# A New Theory to Explain the Underlying Pathogenetic Mechanism of Sudden Infant Death Syndrome

**DOI:** 10.3389/fneur.2015.00220

**Published:** 2015-10-19

**Authors:** Anna Maria Lavezzi

**Affiliations:** ^1^“Lino Rossi” Research Center for the Study and Prevention of Unexpected Perinatal Death and SIDS, Department of Biomedical, Surgical and Dental Sciences, University of Milan, Milan, Italy

**Keywords:** SIDS, pathogenesis, brainstem, respiratory network, fetal breathing, Kölliker–Fuse nucleus, BDNF, NeuN

## Abstract

The author, on the basis of numerous studies on the neuropathology of SIDS, performed on a very wide set of cases, first highlights the neuronal centers of the human brainstem involved in breathing control in perinatal life, with the pontine Kölliker–Fuse nucleus (KFN) as main coordinator. What emerges from this analysis is that the prenatal respiratory movements differ from those post-natally in two respects: (1) they are episodic, only aimed at the lung development and (2) they are abolished by hypoxia, not being of vital importance *in utero*, mainly to limit the consumption of oxygen. Then, as this fetal inhibitory reflex represents an important defense expedient, the author proposes a new original interpretation of the pathogenetic mechanism leading to SIDS. Infants, in a critical moment of the autonomic control development, in hypoxic conditions could awaken the reflex left over from fetal life and arrest breathing, as he did in similar situations in prenatal life, rather than promote the hyperventilation usually occurring to restore the normal concentration of oxygen. This behaviour obviously leads to a fatal outcome. This hypothesis is supported by immunohistochemical results showing in high percentage of SIDS victims, and not in age-matched infant controls, neurochemical alterations of the Kölliker–Fuse neurons, potentially indicative of their inactivation. The new explanation of SIDS blames a sort of auto-inhibition of the KFN functionality, wrongly arisen with the same protective purpose to preserve the life *in utero*, as trigger of the sudden infant death.

## Introduction

### Most Reliable Definition and Hypothesis on SIDS Pathogenesis

The best known definition of SIDS is “the sudden unexpected death of an infant <1 year of age, with onset of the fatal episode apparently occurring during sleep, that remains unexplained after a thorough investigation, including performance of a complete autopsy and review of the circumstances of death and the clinical history” (1). The causes are still unknown, although several even conflicting hypotheses of the underlying mechanisms of SIDS have been proposed (2). The most reliable seems to be the “triple risk hypothesis,” which predicts that fetal brain development of infants who subsequently succumb to SIDS is abnormal, leaving them unable to respond appropriately to stressors during a vulnerable period of the autonomic control (3). Consistent with this assumption, many studies have reported a high incidence of morphological abnormalities and biochemical defects of neurotransmission, particularly serotonergic, in the brainstem of SIDS victims compared with control infants dying of other causes (4, 5). This brain region includes the main nuclei and structures that coordinate the vital activities, such as cardiovascular function and breathing, before and after birth.

### A Rightful Observation

But if the placenta is the effective source of oxygen in fetal life, what is the significance of the respiratory activity in prenatal life? The answer seems to be that the fetus must train so as to be ready to put the lungs to use, once outside the womb. At the time of birth, he will take a few moments to dilate the lungs and begin to breathe. If he fails, survival is threatened. So, *in utero* intermittent breathing movements occur with the main purpose of adequately developing the respiratory system, essential as soon as postnatal life begins (6). The full functionality of the lungs and respiratory muscles acquired will allow the autonomous ventilatory activity that the newborn needs to survive to start up (7).

### Breathing Behavior Before and After Birth in Hypoxic Conditions

A very interesting phenomenon was observed by low-voltage cortical electrical tests in experimental studies on sheep fetuses (8, 9), and also by ultrasound real-time scanning in human fetuses (10): if the amount of oxygen through the placenta decreases (for whatever reason), the fetus immediately suspends pulmonary movements. Precisely, when the partial pressure of oxygen falls below 16–18 mmHg, respiratory activity *in utero* stops, not being a vital function, but only to limit the consumption of oxygen. This is a defense mechanism of the fetus, a way to save energy, because oxygen must go first and foremost to the brain and heart to ensure life.

On the contrary, hypoxia induces hyperventilation in newborns, mainly in the arousal phase from sleep, through a rise in the amplitude and frequency of pulmonary movements, to restore the normal concentration of plasmatic gas and above all of oxygen (11).

### A New Plausible Hypothesis on SIDS Pathogenesis

And it is at this point, on the basis of the above considerations, that I propose a new perspective that could provide a physiological explanation of SIDS. Infants, in a critical moment of the autonomic control development, could awaken a conditioned reflex left over from fetal life: after birth, a situation of lack of oxygen (due to a prone sleep position, nicotine absorption, or any other reason) could induce a vulnerable baby to arrest breathing, he did in similar situations in prenatal life, and, therefore, die. Very probably, being used to being oxygenated by the placenta for 9 months, this ancient instinctive survival behavior could remain registered in the brain, but becoming fatal after birth.

How could the brainstem centers checking the respiratory function be involved in this intrinsic devastating reflex leading to SIDS?

### The Brainstem Respiratory Network

**Figure 1:**
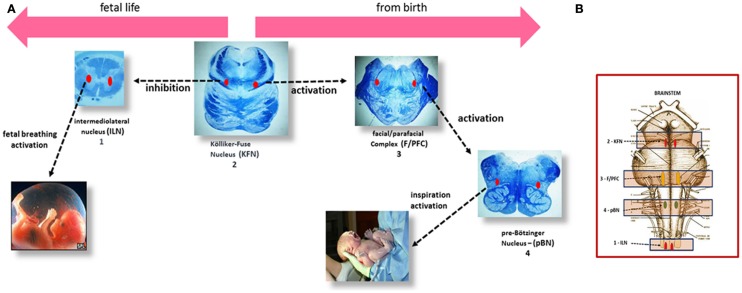
**(A)** Left to right, the steps of the respiratory control in human perinatal life. (1) In the human fetus, episodic respiratory activity aimed at promoting lung development is generated by the intermediolateral nucleus (ILN) in the upper spinal cord. (2) At the same time, during intrauterine life, the Kölliker–Fuse nucleus (KFN), located in the rostral pons, plays an important function by inhibiting the response of central and peripheral chemoreceptors and therefore any respiratory reflex, while allowing the occasional breathing activity headed by the ILN. (3) The facial/parafacial complex (F/PFC), in the caudal pons, starts working at birth, under the stimulation of the KFN, which drastically changes its function, giving rise to the first inspiratory act. The activity of the F/PFC is called “pre-inspiratory” because it is limited to activating, in its turn, the proper inspiratory nucleus in the medulla oblongata: the pre-Bötzinger nucleus (pBN) (4), responsible for starting postnatal breathing. **(B)** Brainstem schematic representation showing the localization of the RN components.

Previous studies, performed at the “Lino Rossi” Research Center of the Milan University, have identified specific nuclei and structures designated to control the breathing, hitherto highlighted only in experimental animals. Given, obviously, the impossibility of performing experiments in humans, the homologous nuclei were identified on the basis of morphological criteria of similarity with regard to the location, the cytoarchitecture, and number of neurons and applying, when possible, immunohistochemical methods to highlight the same neurotransmitters and receptors recognized as specific for several structures, above all in rats. Through this original methodology, the Kölliker–Fuse nucleus (KFN), the facial/parafacial complex (F/PFC), the pre-Bötzinger (pBN) nucleus in the pons/medulla oblongata, and the intermediolateral nucleus (ILN) in the spinal cord were defined (12–15). These nervous centers are linked together via interneuronal synapses in a “respiratory network” (RN), and can modulate one another. I propose a scheme (Figure 1) to illustrate the human breathing control mechanism in perinatal life, indicating the more representative brainstem histological sections where the RN structures are well analyzable, depicted in a chronologically functional sequence, as explained in the legend.

### Role of the Kölliker–Fuse Nucleus in the RN

Essentially, the pulmonary activity is largely dependent on sensory inputs from the ILN in prenatal life and from the F/PFC from birth, being both modulated by the KFN, which therefore represents the breathing filmmaker. Its activity, changing from fetal to postnatal life thanks to a skillful interplay of activation and inactivation of its GABAergic inhibitory and glutamatergic excitatory neurons (16), is fundamental. In hypoxic conditions, a normal fully functional KFN abolishes the rhythmic activity of the ILN in fetuses and greatly accelerates the ventilatory function of the F/PFc in newborns, with the same aim of safeguarding life.

This chief central function exerted by the KFN is supported by experimental studies in fetal lambs performed by brainstem transactions at various levels (17). Results demonstrated the existence of a locus in the rostral lateral pons whose integrity is essential for the depression of breathing during hypoxia *in utero*. The authors suggested that this structure, even if not well-identified anatomically in the sheep brainstem, could correspond to the KFN, previously defined as the core of the “pneuomotaxic center” (18).

### Histological and Immunohistochemical Personal Findings in SIDS

The new etiopathogenic interpretation of SIDS here presented is validated by the findings obtained over many years of personal studies on sudden intrauterine unexplained death syndrome (SIUDS) and SIDS. The “Lino Rossi” Research Center of the Milan University has, in fact, collected a large number of sudden fetal and infant death cases, in application of the Italian Law 31/2006 “*Regulations for diagnostic post mortem investigation in victims of the SIDS and unexpected fetal death*” (19). This law stipulates that all infants who died suddenly in Italian regions within the first year of life and fetuses that die after the 25th week of gestation without any apparent cause, must be rapidly submitted, after obtaining informed parental consent, to in-depth anatomopathological examination, particularly of the autonomic nervous system, with the components of the RN as the main object of study.

Permission from the Ethics Committee was not required for this study as the “Lino Rossi” Research Center is the national referral center for the sudden unexplained fetal and infant deaths, in accordance with the above mentioned Law 31/2006.

**Figure 2:**
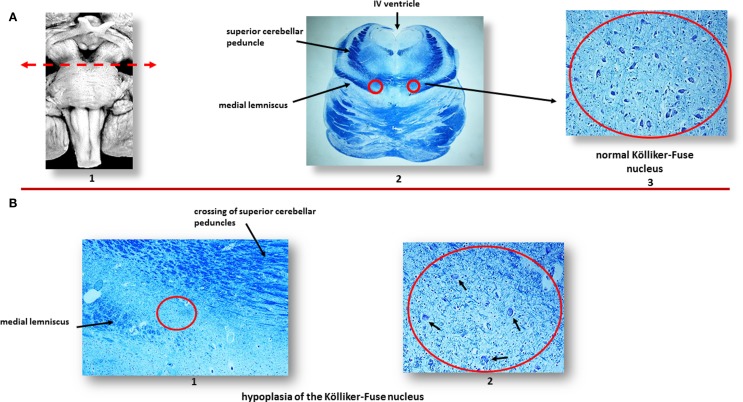
**(A)** Normal Kölliker–Fuse nucleus (KFN) in a control newborn (3-month-old). (1) Brainstem schematic showing the optimal level to examine the KFN (see arrow), though this nucleus is longitudinally extended from the rostral pons to the lower portion of the mesencephalon. (2) Histological transverse section of rostral pons with the indication of the KFN location, between the crossing of the superior cerebellar peduncles and the medial lemniscus. (3) Magnification of the encircled area in (2): the KFN shows its cytoarchitecture consisting of a numerous group of large neurons with distinct, eccentric nucleus, evident nucleolus, and abundant cytoplasm with Nissl substance located at cell periphery. Intermixed with these large neurons, smaller cells (interneurons and astrocytes) are visible. **(B)** Hypoplasia of the KFN in a SIUDS case (39 gestational weeks). The encircled area in (1), included between the crossing of the superior cerebellar peduncles and the medial lemniscus, is represented at higher magnification in (2). In this area, only rare suffering KF neurons are visible (see arrows). Klüver–Barrera staining. Magnification: **(A)** (2): 0.5×; **(A)** (3) 20×; **(B)** (1) 10×; **(B)** (2) 20×.

**Figure 3:**
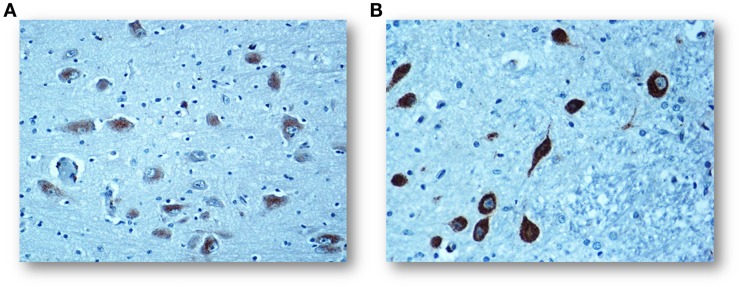
**(A)** Regular negative/weakly immunopositive BDNF expression in the cytoplasm of the KF neurons in a control infant (3-month-old). **(B)** Intense immunopositivity of the KF neurons with dark brown cytoplasmatic staining in a SIDS case, died at 4 months of life. BDNF immunostaining. Magnification: 20×.

**Figure 4:**
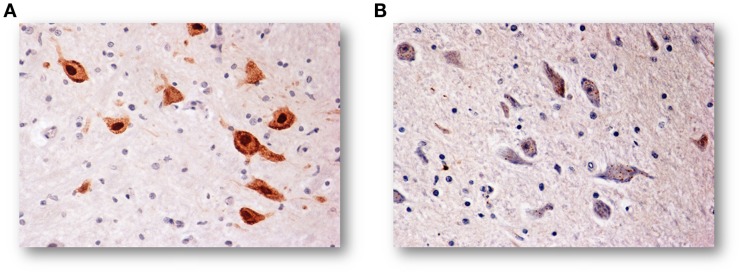
**(A)** NeuN-immunoreactive neurons of the KFN in an infant of the control group (2-month-old). The staining is particularly strong in the neuronal nucleus but also the cytoplasm and the proximal part of the processes are immunoreactive, though to a lesser intensity. **(B)** NeuN-immunonegative KF neurons in an age-matched infant died of SIDS. NeuN immunostaining. Magnification: 20×.

In a large number of SIUDS (up to now no. 95 cases, aged from 25 to 40 gestational weeks), SIDS (no. 150 cases, 1- to 11-month-old), and age-matched controls (no. 35 fetuses and 30 infants), I found developmental hypoplasia of the KFN only in late fetal unexplained deaths (20%), never in newborns and infants (Figure 2) (20). This means that a normal structure of this nucleus is absolutely essential for vital breathing from birth. However, in a high percentage of SIDS cases (no.105, 70%), and not in infant of the control group, despite a normal morphological cytoarchitecture of the KFN, neurochemical alterations, such as an unusual immunopositivity of the brain-derived neurotrophic factor (BDNF) and a decreased expression of the neuronal nuclear antigen (NeuN) were highlighted in the KF neurons (Figures 3 and 4) (21, 22).

The BDNF is a member of the neurotrophin class of growth factors required for the normal development and maturation of specific brainstem centers involved in respiratory control, and therefore expressed in both inhibitory and excitatory neurons of the KFN during the biphasic breathing modulation in intrauterine life (23, 24). After birth, the BDNF is unexpressed in the KFN in human control newborns, while the positivity, as observed in SIDS cases, seems to hinder any ventilatory activity.

By contrast, the NeuN is a protein expressed in post-mitotic functional neurons (25). Then, the specific immunohistochemical method can be applied in neuropathological studies to highlight the physiological status of the neurons (26). While intense NeuN expression is shown by healthy neurons, a considerably reduced NeuN immunopositivity in postnatal life can be indicative of a degeneration of differentiated neurons.

### Correlation of Findings with Nicotine Absorption

In general, morphological and functional brainstem abnormalities resulted significantly related to severe injuries, such as hypoxia. Indeed, the majority of mothers, and often also fathers, of infants with altered KFN development were found to be smokers, either by their own admission, or based on positivity of the cotinine test in the hair of the victims. In case of maternal smoking in pregnancy, carbon monoxide, a gaseous combustion product of nicotine, easily crosses the placental barrier by passive diffusion, and binds to fetal hemoglobin, so giving rise to carboxyhemoglobin (COHb). The same chemical bond occurs when an infant inhales considerable amounts of environmental smoke. COHb is not able to release oxygen into tissues, leading to a general hypoxic status (27). Besides, nicotine is one of the few lipid-soluble substances that can go beyond the blood–brain barrier and induce specific molecular alterations in the DNA, RNA, and antigenic proteins of the nervous cells (28, 29).

## Conclusion

Based on these observations, I propose that during the first months of life, when a predisposed subject is particularly vulnerable, hypoxia can unexpectedly switch on again the ancestral fetal behavior designed to suspend respiration, being this as a non-essential activity, through a functional degeneration of the KF neurons and then a depression of the central respiratory control. This is a protective reflex in the womb but a rapidly fatal device in postnatal life.

Clearly, further studies are required, specifically designed to address this exciting theory that offers consistent assumptions to explain the pathogenetic mechanism occurring in a substantial group of SIDS.

## Conflict of Interest Statement

The author declares that the research was conducted in the absence of any commercial or financial relationships that could be construed as a potential conflict of interest.
